# Electrocardiographic effects of HBI-3000 (sulcardine sulfate), a new drug for termination of atrial fibrillation

**DOI:** 10.1016/j.hroo.2025.11.019

**Published:** 2026-01-02

**Authors:** Jay W. Mason, Suzanne J. Romano, Gary T. Elliott, Boaz Mendzelevski, Stephanie H. Stanworth, Martino Vaglio, Fabio Badilini, Mireille Gillings, Jerome B. Riebman

**Affiliations:** 1 Department of Medicine, University of Utah, Salt Lake City, Utah; 2 HUYABIO International, San Diego, California; 3 Galenic Strategies, San Diego, California; 4 Cardiac Safety Consultants Ltd, London, England, United Kingdom; 5 Stanworth Consulting, Kearns, Utah; 6 AMPS, LLC, New York, New York

**Keywords:** Antiarrhythmic drug, Atrial fibrillation, Cardioversion, Electrocardiogram, Ion channel

## Abstract

**Background:**

HBI-3000 (sulcardine sulfate) inhibits multiple cardiac ion channels in vitro including peak sodium current, late sodium current, L-type calcium current, and the rapid component of the delayed rectifier potassium channel current.

**Objective:**

This study aimed to determine electrocardiographic effects, pharmacokinetics, safety, and tolerability of escalating doses of intravenously administered sulcardine in healthy volunteers.

**Methods:**

In this first-in-human, randomized, double-blind, placebo-controlled, serial-cohort, dose-escalation study, 47 subjects were randomized to 6 cohorts of 8, each receiving 1 of 5 single ascending 30-minute intravenous infusions (20–600 mg) of HBI-3000 (sulcardine sulfate) or placebo in a 6:2 ratio.

**Results:**

Clinically and statistically significant electrocardiographic effects were seen at the higher dose levels (180 mg, 360 mg, and 600 mg). At the probable therapeutic dose (360 mg), concentration-effect modeling predicted the following changes at the time of maximum plasma concentration (30 minutes): Fridericia-corrected QT interval, 13.50 ms; heart rate (HR), 7.70 beats per minute; PR, 17.53 ms; QRS, 7.81 ms; P-wave duration, 9.93 ms; HR corrected J to T peak interval (JTpc), −9.65 ms; and T peak to T end interval, 5.07 ms.

Peak plasma concentrations fell rapidly to negligible levels at 2 hours, associated with rapid redistribution from the central compartment. No significant adverse effects were observed, and no serious adverse events were reported.

**Conclusion:**

Sulcardine increased the QTc and PR intervals, QRS and P-wave durations, and HR dose dependently. The T-wave segment JTpc was significantly decreased, whereas the T peak to T end interval was significantly increased. These findings predict an anti–atrial fibrillation effect via inhibition of 1 or more cardiac ion channels. The strong block of the rapid component of the delayed rectifier potassium channel current was partially mitigated by JTp shortening, probably owing to late sodium current and L-type calcium current inhibition, reducing the risk of proarrhythmia by decreasing repolarization time.

**Key Findings**

▪ HBI-3000 (sulcardine sulfate) is a new intravenous (IV) antiarrhythmic drug for the treatment of recent-onset atrial fibrillation.
▪ HBI-3000 blocks multiple cardiac ion channels including peak sodium current, late sodium current (I_Na-late_), L-type calcium current (I_Ca,L_), and the rapid component of the delayed rectifier potassium channel current (I_Kr_).
▪ Excessive QT prolongation by HBI-3000 by block of I_Kr_ is prevented by its inhibition of I_Na-late_ and I_Ca,L_, which shortens the J to T peak interval, reducing the overall extent of QT prolongation.
▪ HBI-3000 was well tolerated up to a dose of 600 mg IV over 30 minutes.

## Introduction

HBI-3000 [the sulfate salt of sulcardine, N-(4-hydroxy-3,5-bis(pyrrolidin-1-ylmethyl)benzyl)-4 methoxybenzene sulfonamide sulfate] is a novel multi-ion channel blocker being evaluated as a short-term intravenous (IV) infusion for cardioversion of atrial fibrillation (AF).^1^ The drug originated at the Shanghai Institute of Materia Medica, Shanghai, China, where it is being evaluated in human clinical trials as an oral treatment for premature ventricular beats.^2^, ^3^, ^4^, ^5^, ^6^, ^7^ Sulcardine is a synthetic analog designed to improve the physical properties and safety profile of changrolin, an antiarrhythmic molecule derived from an herbal traditional Chinese medicine originally used to treat malaria and found to have ventricular and atrial antiarrhythmic effects, including efficacy against AF.^2^^,^^8^, ^9^, ^10^

In vitro pharmacology studies in failing and nonfailing human ventricular cardiomyocytes^11^ showed that sulcardine blocked multiple cardiac ion channels (peak sodium current [I_Na-peak_], late sodium current [I_Na-late_], L-type calcium current [I_Ca,L_], and rapid component of the delayed rectifier potassium channel current [I_Kr_]) at relevant concentrations, as well as early after-depolarizations induced by heart failure or dofetilide, some or all of which may be involved in the initiation and maintenance of acute-onset AF. In a canine sudden-death model of regional myocardial ischemia after antecedent infarction, sulcardine showed improved safety vs flecainide.^12^ Herein, we report results of the first-in-human dose-escalation study of an IV formulation of sulcardine.

## Methods

### Investigative site

The study, HBI-3000-301 (NCT03397641), was conducted at Quotient Sciences (Nottingham, United Kingdom), which was also responsible for the manufacture of HBI-3000 (sulcardine sulfate) drug product for IV administration. Enrolled subjects were confined in the clinical pharmacology unit throughout the duration of the study. The study was approved by a local ethics committee (South Central – Berkshire Research Ethics Committee, Bristol, United Kingdom). Each participating subject signed a consent form approved by the ethics committee. This research study adhered fully to the Consolidated Standards of Reporting Trials and the Declaration of Helsinki guidelines.

### Subjects

Study participants (N = 47) were recruited from a healthy volunteer database maintained by the clinical pharmacology unit. Major exclusion criteria were presence or history of cardiovascular disease, cardiac arrhythmias, cardiac conduction abnormalities, Fridericia-corrected QT interval (QTcF) of >450 ms, and QRS of >120 ms. 47 subjects were randomized in cohorts of 8 to receive active drug or placebo in a 6:2 ratio at sequential doses of 20, 60, 180, 360, and 600 mg HBI-3000. (These doses, which are used throughout the text, were calculated as the sulfate salt of sulcardine [HBI-3000] and are equivalent to 16.5, 50, 149, 298, and 496 mg sulcardine free base, respectively.) The 600 mg dose was administered to 2 cohorts for a total of 12 receiving active drug at that dose. The 360 mg cohort had only 7 subjects, given that neither the scheduled nor the alternate subjects were available to complete it. As a safety precaution, in each cohort where an increased dose was administered, 2 sentinel subjects (1 active to 1 placebo) were dosed at least 22 hours ahead of the main group; the remaining 6 subjects were dosed in a 5:1 ratio of active to placebo. The principal investigator assessed safety and tolerability before the remaining subjects continued with dosing.

### Study design

This was a randomized, double-blind, placebo-controlled, single ascending dose study. Consenting adults aged 18–50 years were screened within 28 days of entering the clinical pharmacology unit 2 days before drug administration (day −2) and were confined until discharge on the morning of day 4. Placebo or active drug was administered on the morning of day 1 as a 30-minute, fixed-dose IV infusion. The electrocardiogram (ECG) was continuously recorded by a 12-lead Holter monitor from 24 hours before dosing to 48 hours after dosing. Triplicate 10-second, 12-lead ECGs were extracted from the Holter recordings. Blood samples for plasma concentration determination were obtained at the following time points: immediately before dosing and 0.25 (halfway through the infusion), 0.5 (end of the infusion), 1, 2, 3, 4, 6, 8, 12, and 24 hours after initiation of the IV infusion.

### Pharmacokinetic sample acquisition and analysis

Pharmacokinetic (PK) venous blood samples were drawn immediately after programmed ECG extraction time points. The plasma was preserved in K_2_-EDTA, frozen within 1 hour, stored at −20°C, and shipped to LGC Ltd (Teddington, United Kingdom) for analysis. Plasma sample analysis for sulcardine was performed using high-performance liquid chromatography with a C18 reversed-phase column, and mass spectral analysis detection (positive-ion mode) was done using a deuterated internal standard (HBI-3000-d8) and a gradient mobile phase of neat acetonitrile and 10 mM ammonium formate, pH 3.

### ECG acquisition and analysis

ECG equipment and acquisition were managed by a core ECG laboratory (Biomedical Systems). ECGs were extracted in triplicate, at least 1 minute apart, from a 5-minute prespecified time window at the nominal time points listed earlier from a Mortara H12+ recording. Blinded cardiologists at Biomedical Systems overread interpretable ECGs, and these readings were used to identify treatment-emergent diagnostic statement abnormalities.

Many ECG recordings were affected by 50 Hz AC interference at the investigative site, rendering them unmeasurable, uninterpretable, or unextractable. Consequently, all ECGs were extracted again by AMPS LLC after application of 2 separate filters applied in cascade: (1) an adaptive filter for line-interference removal based on a 50th-order least mean squares line-enhancement algorithm tuned to a 50 Hz component and (2) a low-pass filter based on a bidirectional recursive fifth-order Butterworth filter with a cutoff frequency set at 50 Hz. An unfiltered dataset was also generated.

After filtering, PR, QRS, QT intervals, P-wave duration (P_Dur_), and additional T-wave segmentation parameters (J to T peak interval [JTp] and T peak to T end interval [TpTe]) were automatically measured on the newly created noise-free, global-superimposed median beats by the AMPS Bravo algorithm. JTp is the T-wave segment from the J point to the highest peak of the T wave, and JTp, heart rate (HR) corrected (JTpc), is JTp corrected for HR using the method of Johannesen et al.^13^ *JTpc* = *JTp* / (*RR*)0.58, and TpTe is the T-wave interval from the highest peak of the T wave to the end of the T wave. Mean values for HR and the PR, P_Dur_, QRS, QT, QTc, JTpc, and TpTe intervals were calculated from the denoised triplicate data for statistical analysis. The Fridericia correction for the QT interval at HR was used. The results reported herein and in Supplemental Section A are based on the denoised ECG data. The effect of denoising on ECG interval values was assessed by comparing filtered and unfiltered ECG data and was found to be minimal (Supplemental Section B).

### Muscarinic receptor binding assays

These assays were performed to determine possible causes of the HR increase observed in this study after sulcardine administration. In a broad screen for off-target interactions using a commercially available in vitro panel of enzymes, ion channels, receptors, and transporters, sulcardine displayed some inhibition of human M_2_ muscarinic acetylcholine receptor [M_2_(h)] activity. Follow-up in vitro antagonist radioligand binding assays and cellular receptor functional assays for M_2_(h) (source: human recombinant Chinese hamster ovary cells) were performed by Eurofins using 9 concentrations of sulcardine (tested as HBI-3000) ranging from 10 nM to 100 μM, according to their standard procedures. Methoctramine served as the target-specific ligand.

### Statistical analysis

The study was exploratory, and no formal sample size calculation was made. Based on experience from previous studies with a similar design and purpose, a total of 8 subjects were enrolled per cohort, and a minimum of 6 evaluable subjects was considered sufficient. An evaluable subject was defined as a subject who had completed all or most of the safety and PK assessments up to 72 hours after the dose.

Mean baseline-adjusted (Δ) and baseline + placebo–adjusted (ΔΔ) ECG values were analyzed using concentration-effect modeling of each plasma concentration–mean triplicate ECG data pair to predict ECG interval changes at the observed maximum plasma concentration (C_max_) for each dose level. The mixed-effects model included ΔQTcF as the dependent variable and corresponding sulcardine plasma concentrations, treatment (active or placebo), time point, and baseline QTcF as independent variables. Plasma concentration values that were below the limit of quantitation and placebo treatment concentrations were set to 0. A quadratic concentration term was added to the model and kept if the slope was significantly different from 0 and the Akaike’s information criterion was smaller than the linear model. Outlier analysis of ECG data was performed using the categorical criteria listed in Supplemental Section A (Supplemental Table A1).

The frequency of treatment-emergent diagnostic statement abnormalities (N subjects with abnormality divided by all N subjects) was listed by treatment and dose for each time point for any abnormality not present at baseline that appeared on any of the triplicate ECGs. For this presentation, diagnostic abnormalities over all on-treatment time points were compared for each sulcardine dose and placebo.

## Results

### Subject demographics and disposition

The mean age of the 47 enrolled subjects was 32.9 ± 9.2 years (standard deviation). The age range was 20–50 years, and 30 (64%) were males. Racial distribution was predominantly white (87%). All 47 randomized subjects received their full treatments and completed the study.

### PK

**Figure 1:**
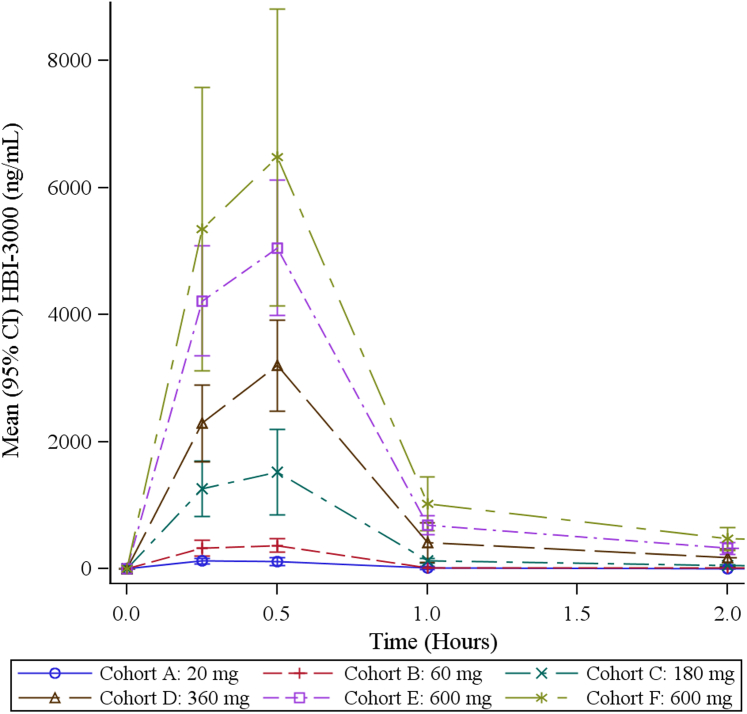
Sulcardine plasma concentration. Observed plasma concentrations of sulcardine sulfate over time after initiation of the 30-minute, fixed-dose infusion are displayed. The insert shows the first hour with the time scale expanded. The plasma concentration profile was dose proportional. CI = confidence interval; ng/mL = nanograms per milliliter.

Plasma concentration of sulcardine during the infusion and the subsequent 24 hours is presented in Figure 1. The figure insert shows that the time of C_max_ occurred at the end of the infusion (0.5 hour) at all dose levels and that concentrations were dose proportional. Concentrations fell to negligible levels (approximately 4% to 6% of C_max_) at all doses by 2 hours. Selected PK parameters at each dose level are presented in Table 1.

**Table 1:** Selected pharmacokinetic data

Dose of HBI-3000 (sulcardine sulfate)	N	T_max_ (h) (min – max)	C_max_ (ng/mL) [G. CV (%)]	AUC_(0-24)_ (ng·h/mL) [G. CV (%)]	AUC_(0-last)_ (ng·h/mL) [G. CV (%)]	AUC_(0-inf)_ (ng·h/mL) [G. CV (%)]	CL (mL/min) [G. CV (%)]	V_ss_ (L) [G. CV (%)]	V_z_ (L) [G. CV (%)]
20 mg	6	0.42	131	89.7	86.2	175	1920	73.2	103
		(0.25–0.52)	(29.60)	(53)	(54.80)	(NC)	(NC)	(NC)	(NC)
60 mg	6	0.38	369	299	306	312	3260	586	2580
		(0.25–0.52)	(23.80)	(17.40)	(18.30)	(NC)	(NC)	(NC)	(NC)
180 mg	6	0.46	1430	1270	1550	2050	1460	2440	6680
		(0.25–0.50)	(41.60)	(25.60)	(23)	(41.80)	(41.70)	(10.40)	(4.30)
360 mg	5	0.45	3170	2960	3470	3830	1600	1810	5000
		(0.27–0.50)	(17.60)	(23.70)	(26.60)	(33.80)	(33.60)	(18.30)	(33.10)
600 mg	12	0.46	5580	5810	6640	7580	1310	1490	4670
		(0.25–0.50)	(30.50)	(31.20)	(30.50)	(28.90)	(28.70)	(32.50)	(32.30)

AUC_(0-24)_ = area under the concentration vs time curve between 0 hours and 24 hours; AUC_(0-inf)_ = area under the concentration vs time curve between 0 hours and extrapolation to infinity; AUC_(0-last)_ = area under the concentration vs time curve between 0 hours and last sample collection time measurement; CL = clearance rate; C_max_ = maximum plasma concentration; min = minimum; max = maximum; G. CV = geometric coefficient of variation; NC = not calculated; T_max_ = time of maximum plasma concentration; V_ss_ = volume of distribution at steady state after intravenous dosing; V_z_ = apparent volume of distribution during the terminal elimination phase.

### ECG

The primary analysis was concentration-effect modeling from which Δ and ΔΔECG changes were predicted.

### Concentration-effect modeling

**Figure 2:**
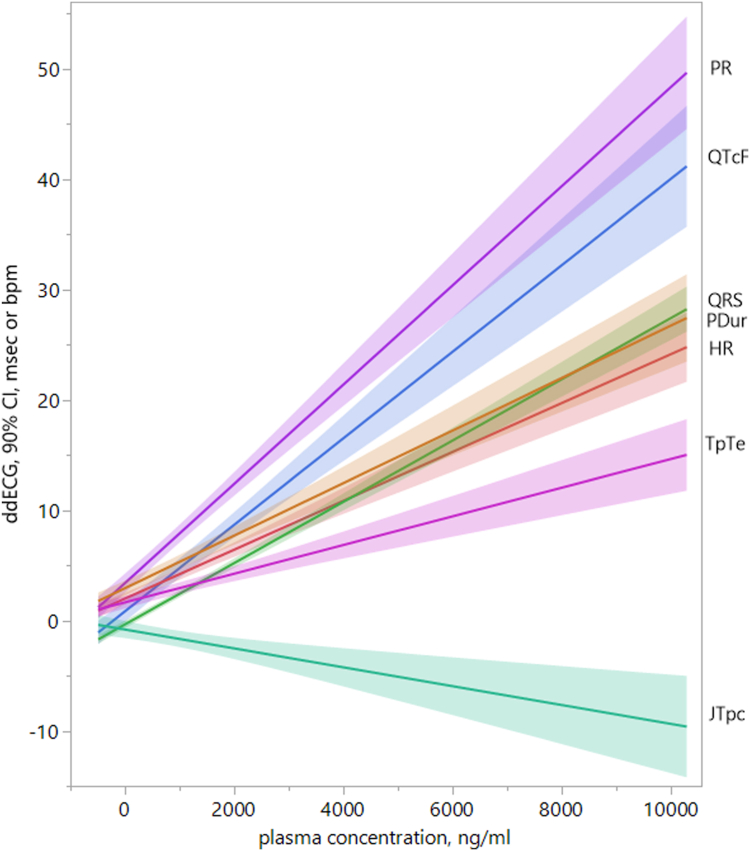
Sulcardine concentration—ECG effect. Simple, algebraic linear fits and 90% CIs of heart rate and ECG intervals (QTcF, QRS, PR, P_Dur_, JTpc, and TpTe) to plasma concentrations including all doses of sulcardine are shown. Dose-related increases of heart rate, QTcF, QRS, PR, P_Dur_, and TpTe were observed, whereas a dose-related decrease in JTpc was observed, consistent with sulcardine’s known inhibition of I_Na-peak_, I_Na-late_, I_Ca,L_, and I_Kr_ at therapeutic concentrations. bpm = beats per minute; CI = confidence interval; ECG = electrocardiogram; ddECG = baseline and placebo-corrected ECG parameter; HR = heart rate; I_Ca,L_ = L-type calcium current; I_Kr_ = rapid component of the delayed rectifier potassium channel current; I_Na-late_ = late sodium current; I_Na-peak_ = peak sodium current; JTpc = J to T peak interval, HR corrected; msec = milliseconds; P_Dur_ = P-wave duration; PR = PR interval; QRS = QRS interval; QTcF = Fridericia-corrected QT interval; TpTe = T peak to T end interval.

Simple linear algebraic fits for each HR and ECG interval to the plasma concentration of sulcardine are presented in Figure 2. Concentration-mixed effect models were performed individually for each ECG parameter (HR, PR, P_Dur_, QRS, QTcF, JTpc, and TpTe). The plots of each analysis showing ΔECG vs plasma concentrations of sulcardine are displayed in the Supplemental Section A. ΔΔECG, the on-treatment ΔECG minus the placebo ΔECG, was then calculated using the concentration-effect model results. Predictions of the resulting ΔΔECG at the observed C_max_ at each dose level are presented in Table 2. The concentration-effect relationships were statistically significant for all ECG variables, and the slopes were all positive except for that of ΔΔJTpc. A well-behaved dose-response relationship was present for all ECG variables, with the exception that predicted JTpc reduction was greatest at the 360 mg level rather than at 600 mg. The models predict measurable ECG effects at 180 mg, becoming substantial at higher doses, and predict an increase in HR and in repolarization (QTcF), atrioventricular conduction (PR), intra-atrial conduction (P_Dur_), and intraventricular conduction (QRS) times. What might have been a larger increase in QTcF was mitigated by an actual shortening of JTpc.

**Table 2:** Concentration-effect model predictions and parameters

C_max_ (dose)	Mean 90% CI						
	ΔΔQTcF, ms	ΔΔHR, bpm	ΔΔPR, ms	ΔΔQRS, ms	ΔΔP_Dur_, ms	ΔΔJTpc, ms	ΔΔTpTe, ms
135 ng/mL (20 mg)	1.40 −2.085 to 4.878	1.74 −0.528 to 4.017	5.08 1.409–8.744	−0.08 −1.530 to 1.373	3.17 −0.513 to 6.857	−0.59 −4.129 to 2.953	1.95 −0.478 to 4.376
378 ng/mL (60 mg)	2.35 −1.121 to 5.827	2.21 −0.051 to 4.480	6.06 2.405–9.716	0.564 −0.904 to 1.993	3.71 0.028–7.384	−1.81 −5.347 to 1.719	2.20 −0.226 to 4.618
1530 ng/mL (180 mg)	6.89 3.386–10.388	4.44 2.159–6.730	10.73 7.029–14.422	3.50 2.036–4.960	6.24 2.532–9.944	−6.43 −10.209 to −2.661	3.37 0.925–5.805
3120 ng/mL (360 mg)	13.50 9.778–17.220	7.70 5.230–10.162	17.53 13.502–21.557	7.81 6.245–9.366	9.93 6.018–13.850	−9.65 −13.910 to −5.387	5.07 2.490–7.651
5280 ng/mL (600 mg)	23.77 19.368–28.176	12.75 9.727–15.769	28.10 23.082–33.116	14.50 12.636–16.359	15.67 11.09–20.25	−6.34 −10.916 to −1.773	7.72 4.698–10.742
Slope	0.0039	0.0019	0.0040	0.0026	0.0022	−0.0054	0.0010
*P* value	<.0001	<.0001	<.0001	<.0001	<.001	<.0001	<.0001

ΔΔ = baseline and placebo corrected; bpm = beats per minute; CI = confidence interval; C_max_ = maximum plasma concentration; HR = heart rate; JTpc = J to T peak interval, HR corrected; P_Dur_ = P-wave duration; PR = PR interval; QRS = QRS interval; QTcF = Fridericia-corrected QT interval; TpTe = T peak to T end interval.

### Outlier responses

As expected, sulcardine resulted in categorical increases in all standard ECG intervals, increased HR, and increased atrial and ventricular conduction times, whereas placebo recipients showed little change. Categorical changes for all ECG variables are summarized in the Supplemental Section A.

Although no case of QTcF of >450 ms occurred in the placebo group (N = 12), 3 cases of QTcF of >450 ms were seen in the sulcardine groups combined (N = 35); there were no instances of QTcF of >500 ms. These 3 subjects, consisting of 1 male and 2 females, had all received 600-mg doses of HBI-3000. The highest observed QTcF among the 3 subjects was 484 ms at 0.5 hours (increased from 437 ms) in a female subject. A change from baseline of ≥30 ms and <60 ms was observed in 7 of the 35 subjects (4 females and 3 males) receiving sulcardine, all of whom received 600 mg; none were observed in the placebo group. A change from baseline of >60 ms (63.1 ms) was seen in 1 female subject receiving 600 mg. These categorical differences from placebo are consistent with the identified QTc-prolonging effect of sulcardine, but extreme changes were reassuringly absent.

An HR of <50 beats per minute (bpm) with a 25% or greater decrease from baseline was not observed in this study. The lowest observed HR was 41 bpm at 3 hours in a subject receiving 600 mg. HR of >100 bpm with a 25% or greater increase over baseline occurred in 1 subject receiving 600 mg at 0.75 hours after dosing. The highest observed HR was 105 bpm at 0.75 hours in a subject receiving 600 mg.

PR of >200 ms with a 25% or greater increase over baseline was seen 4 times in the sulcardine recipients: 1 subject receiving 60 mg at 1 hour, 1 subject receiving 360 mg at 0.75 hour, 2 subjects receiving 600 mg at 0.5 hour, and 0 times in placebo subjects. The highest observed PR was 214 ms at 0.5 hours in a subject receiving 180 mg sulcardine.

QRS of >100 ms with a 25% or greater increase over baseline occurred 3 times in subjects receiving 600 mg and none in placebo recipients. The highest observed QRS was 130 ms (increased from 104 ms) at 0.5 hours in a subject receiving 600 mg. This same subject had the highest observed QTcF at the same time point.

### Diagnostic statement abnormalities

There were 9 specific treatment-emergent ECG diagnostic statement abnormalities. The frequency of their occurrences is presented in Table 3. The events were similarly infrequent at all dose levels including placebo, and none of these ECG events were clinically worrisome. All were expected based on the ion channel inhibition profile of sulcardine.

**Table 3:** Treatment-emergent ECG diagnostic abnormalities

Diagnostic terms	20 mg	60 mg	180 mg	360 mg	600 mg	600 mg	Combined	Placebo
n	6	6	6	5	6	6	35	12
First-degree AV block	0 (0.0%)	0 (0.0%)	0 (0.0%)	0 (0.0%)	0 (0.0%)	2 (33.3%)	2 (5.7%)	0 (0.0%)
Intraventricular conduction delay	0 (0.0%)	0 (0.0%)	1 (16.7%)	0 (0.0%)	1 (16.7%)	0 (0.0%)	2 (5.7%)	0 (0.0%)
Left axis deviation	0 (0.0%)	1 (16.7%)	0 (0.0%)	0 (0.0%)	0 (0.0%)	1 (16.7%)	2 (5.7%)	0 (0.0%)
Incomplete right bundle branch block	0 (0.0%)	0 (0.0%)	0 (0.0%)	0 (0.0%)	0 (0.0%)	1 (16.7%)	1 (2.9%)	0 (0.0%)
Nonspecific T-wave abnormality	1 (16.7%)	0 (0.0%)	0 (0.0%)	0 (0.0%)	1 (16.7%)	0 (0.0%)	2 (5.7%)	0 (0.0%)
Premature atrial complexes	1 (16.7%)	0 (0.0%)	0 (0.0%)	0 (0.0%)	0 (0.0%)	0 (0.0%)	1 (2.9%)	0 (0.0%)
Right axis deviation	1 (16.7%)	0 (0.0%)	2 (33.3%)	1 (20.0%)	1 (16.7%)	4 (66.7%)	9 (25.7%)	4 (33.3%)
Sinus bradycardia	2 (33.3%)	2 (33.3%)	4 (66.7%)	1 (20.0%)	2 (33.3%)	1 (16.7%)	12 (34.3%)	4 (33.3%)
Sinus tachycardia	0 (0.0%)	0 (0.0%)	0 (0.0%)	0 (0.0%)	0 (0.0%)	1 (16.7%)	1 (2.9%)	0 (0.0%)

AV = atrioventricular; ECG = electrocardiogram.

### Influence of denoising on ECG data

As indicated earlier, 50 Hz AC interference affected many ECGs extracted from the continuous 12-lead Holter recording, which served as the primary source of ECG data in this study. Electronic filters (a 50 Hz notch filter and a 50 Hz bandpass filter) were used to denoise the recordings and were independently tested. A description of that process and an analysis of its effects on ECG data are presented in the Supplemental Section B. Denoising was successful and did not significantly affect the ECG analysis.

### Changes in blood pressure

A slight, dose-related decrease in systolic blood pressure was observed in sulcardine recipients, exceeding that in placebo recipients by a group-mean maximum of −3.6 mm Hg at the end of the infusion. This decrease was associated with increased HR and diastolic blood pressure, resulting in a reduction in pulse pressure while maintaining mean blood pressure, and, therefore, may not indicate negative inotropy. The HR and blood pressure changes are displayed and analyzed in the Supplemental Section C. It is noteworthy that a reduction in left ventricular ejection fraction was not observed at any dose of sulcardine in a recent study of patients with AF undergoing pharmacologic conversion to sinus rhythm.^1^

### Adverse effects

A total of 3 subjects (25.0%) receiving placebo and 13 (37.1%) receiving active treatment reported at least 1 treatment-emergent adverse event (TEAE) with 2 (33.3%), 2 (33.3%), 0 (0.0%), 2 (40.0%) and 7 (58.3%) in the 20 mg, 60 mg, 180 mg, 360 mg, and 600 mg HBI-3000 groups, respectively.

Headache (4 [11.4%]) and back pain (3 [8.6%]) were the most commonly reported TEAEs in the active groups, followed by dysgeusia (2 [5.7%]) and rash at the medical device (ECG electrode) site (2 [5.7%]). The subject incidence of reported TEAEs was dose related, and there was a higher incidence of TEAEs at the 600 mg dose of HBI-3000 than the other active treatment doses. Dysgeusia (metallic taste), paresthesia, and oral hypesthesia, reported only by HBI-3000–treated subjects at the highest (600 mg) dose level, at the approximate time of maximal exposure (30 minutes), may be considered indicative of the pharmacologic effect of sulcardine, related to I_Na-peak_ block.

### Muscarinic receptor binding assay

In the in vitro binding assays, the half-maximal inhibitory concentration (IC_50_) and K_i_ of sulcardine for M_2_(h) were 28 μM and 20 μM, respectively. In the functional assays, these values were 78 μM and 9.1 μM, respectively. These IC_50_ values are in the same range as sulcardine inhibition of its target ion channels (Table 4). The active control, methoctramine, had an IC_50_ of 41 nM and a K_i_ of 29 nM in the binding assays and 210 nM and 25 nM, respectively, in the intact-cell-based functional assays.

**Table 4:** Relative inhibitory potencies (IC_50_, μM) of sulcardine (as HBI-3000) on human ventricular myocardial ion channels^11^

IC_50_ (μM)			
I_Na-peak_	I_Na-late_	I_Kr_	I_Ca,L_
48.3 ± 3.8	16.5 ± 1.4	22.7 ±2.5	32.2 ± 2.9

IC_50_ = half-maximal inhibitory concentration; I_Ca,L_ = L-type calcium current; I_Kr_ = rapid component of the delayed rectifier potassium channel current; I_Na-late_ = late sodium current; I_Na-peak_ = peak sodium current.

## Discussion

The IV formulation of HBI-3000 (sulcardine sulfate), in development for the termination of acute AF, was found to be safe and well tolerated, with potent ECG effects, in this study.

### Summary of clinical results

The primary analysis in this study, concentration-effect modeling of the ECG effects of sulcardine, showed substantial dose- and concentration-related increases of HR, PR, P_Dur_, QRS, and QTcF. T-wave segment analysis showed a decrease in JTpc and an increase in TpTe. Categorical ECG changes were consistent with these observations. All findings were expected based on preclinical studies. Adverse effects were minimal and benign. There was no evidence for a clinically significant hemodynamic effect. Treatment-emergent ECG diagnostic abnormalities were clinically mild and unremarkable.

### Ion channel effects of sulcardine

The human ventricular ion channel profile of sulcardine was described by Guo et al^11^ using in vitro voltage clamp protocols in single human cardiomyocytes, as shown in Table 4, wherein similar IC_50_ values are documented for I_Na-peak_, I_Na-late_, I_Kr_, and I_Ca,L_. Our observations in the current study are fully consistent with those channel interactions: I_Na-peak_ block could account for both QRS and P-wave widening, as well as PR prolongation; I_Ca,L_ block could contribute to both PR prolongation and JTpc shortening; I_Na-late_ block could contribute to JTpc shortening; and I_Kr_ block could account for both QTcF and TpTe prolongation. Isolated I_Kr_ block typically prolongs the 2 T-wave segments (JTpc and TpTe) equally.^13^ Complete reversal of its effect on JTpc, with actual shortening of that interval, was probably owing to a combination of I_Na-late_ block and I_Ca,L_ block, because reduction, but not reversal, of I_Kr_ block-induced JTpc prolongation is usually seen with inhibition of either channel alone. JTpc shortening would be expected to protect against excessive QT prolongation and torsades de pointes ventricular tachycardia. A similar extent of JTpc shortening by sulcardine was again observed in 2 recent studies reported in abstract form.^1^^,^^14^

Drugs that primarily affect 1 or more of the channels blocked by sulcardine are capable of terminating AF. These include numerous I_Na-peak_ blockers, such as quinidine,^15^ flecainide,^16^ and amiodarone^17^; I_Kr_ blockers, such as ibutilide,^18^ dofetilide,^19^ and amiodarone^17^; and I_Na-late_ blockers, such as ranolazine.^20^ It is also known that inhibition of I _Na-late_^21^ and of I_Ca,L_^22^^,^^23^ shortens the JTpc and, as a result, the QTc intervals when prolonged by an I_Kr_ blocker, thereby reducing the risk of torsades de pointes. In a recent clinical trial, reported in abstract form, sulcardine was shown to be effective in patients with AF of recent onset,^19^ with a conversion rate of 69% in nonobese patients at the likely therapeutic dose range of 200–500 mg HBI-3000 IV. It seems likely that sulcardine’s channel-blocking profile will be capable of terminating recent-onset AF with a minimized risk of I_Kr_-related proarrhythmia.

The mechanism responsible for the dose-related increase in HR by sulcardine does not seem to be a reflex response to hemodynamic impairment, given that mean blood pressure was unaltered. The anticholinergic effect of sulcardine, as observed in receptor binding and functional assays, and possibly the expected baroreflex effect of arterial I_Ca,L_ block may explain the transient increase in HR observed in the clinical trial.

In this phase 1 clinical study, the modest decline in systolic BP without a change in mean BP in normal subjects may suggest that there is no significant adverse inotropic effect or that physiological mechanisms readily compensate for any inotropic effect.

### Study limitations

This first-in-human study was necessarily limited to healthy subjects in normal sinus rhythm. The antiarrhythmic effect of sulcardine against AF has been demonstrated in a small open-label phase 2 trial,^1^ but not yet in a large phase 3 trial.

A major shortcoming of most antiarrhythmic drugs used to terminate AF is their potential to cause or worsen heart failure or to be proarrhythmic. Although this study did not find evidence of hemodynamic liability resulting from sulcardine exposure, its hemodynamic effects in humans must be further explored. Although the dose-related decrease in the JTpc is suggestive of reduced proarrhythmic liability from excessive QT prolongation and no such arrhythmia occurred in a recent study in continuously monitored patients with AF,^1^ additional clinical studies will be needed to explore this further. The effects of structural cardiac abnormalities and electrical remodeling on the clinical use of sulcardine in patients are unknown. However, its protective repolarization properties should prevent the proarrhythmic effects of sodium channel blockers observed in the CAST trial.^24^

Some ECGs in this study were rendered unmeasurable or uninterpretable by 50 Hz AC interference at the investigative site. However, electronic filtering of the ECG signals mitigated the problem with no negative consequences (Supplemental Section B).

## Conclusion

Sulcardine blocks multiple cardiac ion channels with demonstrable ECG changes. These effects correspond with its established, targeted ion channel interactions and characteristics of other known anti-AF drugs. Safety of sulcardine for termination of acute AF and treatment of other arrhythmias may be enhanced by its shortening of JTpc, reducing the risk of QT prolongation-related arrhythmias, and its increase in HR may reduce the risk of postconversion sinus bradycardia. Results from an open-label phase 2 study showing safety and efficacy of a single IV infusion of sulcardine (as HBI-3000) for the conversion of AF of recent onset (HBI-3000-402, NCT#04680026) have been reported in abstract form.^1^

## Disclosures

Gillings, Romano, and Riebman were employees of HUYABIO International. Mason, Elliott, and Mendzelevski were consultants to HUYABIO International. Stanworth, Vaglio, and Badilini were vendors to HUYABIO International.

## Footnotes

Appendix Supplementary data associated with this article can be found in the online version at https://doi.org/10.1016/j.hroo.2025.11.019.
