# Supplementary material for: Electrocardiographic effects of HBI-3000 (sulcardine sulfate), a new drug for termination of atrial fibrillation
Source: Heart Rhythm O2. 2026 Jan 2;7(2):264–72. doi: 10.1016/j.hroo.2025.11.019 (PMC12925892; doi:10.1016/j.hroo.2025.11.019)
Supplement: Supplementary File [file mmc1.docx]

**Online Supplemental Material to**

**Electrocardiographic Effects of Sulcardine, a New Drug for Termination of Atrial Fibrillation**

**Sections A, B and C**

**Supplement**

**Section A**

**Categorical Change in ECG Variables during Treatment and**

**ΔECG Concentration-Effect Model Plots**

**Table A1: Summary Statistics of Categorical QTcF Interval (ECG Population), msec**

| **Time Point  QTcF Interval, CFB** | **Cohort A 20 mg** | **Cohort B 60 mg** | **Cohort C 180 mg** | **Cohort D 360 mg** | **Cohort E 600 mg** | **Cohort F 600 mg** | **Combined HBI-3000** | **Placebo** | |
| --- | --- | --- | --- | --- | --- | --- | --- | --- | --- |
| **Maximum QTcF value post dose** | | | | | | | | | |
| QTcF ≤ 450 msec | 5 (83.3%) | 6 (100.0%) | 6 (100.0%) | 5 (100.0%) | 5 (83.3%) | 4 (66.7%) | 31 (88.6%) | 12 (100.0%) | |
| QTcF > 450 msec | 1 (16.7%) | 0 (0.0%) | 0 (0.0%) | 0 (0.0%) | 1 (16.7%) | 1 (16.7%) | 3 (8.6%) | 0 (0.0%) | |
| QTcF > 480 msec | 0 (0.0%) | 0 (0.0%) | 0 (0.0%) | 0 (0.0%) | 0 (0.0%) | 1 (16.7%) | 1 (2.9%) | 0 (0.0%) | |
| QTcF > 500 msec | 0 (0.0%) | 0 (0.0%) | 0 (0.0%) | 0 (0.0%) | 0 (0.0%) | 0 (0.0%) | 0 (0.0%) | 0 (0.0%) | |
| CFB ≤ 30 msec | 5 (83.3%) | 6 (100.0%) | 6 (100.0%) | 4 (80.0%) | 3 (50.0%) | 0 (0.0%) | 24 (68.6%) | 12 (100.0%) | |
| CFB > 30 and ≤ 60 msec | 1 (16.7%) | 0 (0.0%) | 0 (0.0%) | 1 (20.0%) | 3 (50.0%) | 5 (83.3%) | 10 (28.6%) | 0 (0.0%) | |
| CFB > 60 msec | 0 (0.0%) | 0 (0.0%) | 0 (0.0%) | 0 (0.0%) | 0 (0.0%) | 1 (16.7%) | 1 (2.9%) | 0 (0.0%) | |
| **0.25 h** | | | | | | | | | |
| QTcF ≤ 450 msec | 5 (83.3%) | 6 (100.0%) | 6 (100.0%) | 5 (100.0%) | 6 (100.0%) | 5 (83.3%) | 33 (94.3%) | 12 (100.0%) | |
| QTcF > 450 msec | 1 (16.7%) | 0 (0.0%) | 0 (0.0%) | 0 (0.0%) | 0 (0.0%) | 1 (16.7%) | 2 (5.7%) | 0 (0.0%) | |
| QTcF > 480 msec | 0 (0.0%) | 0 (0.0%) | 0 (0.0%) | 0 (0.0%) | 0 (0.0%) | 0 (0.0%) | 0 (0.0%) | 0 (0.0%) | |
| QTcF > 500 msec | 0 (0.0%) | 0 (0.0%) | 0 (0.0%) | 0 (0.0%) | 0 (0.0%) | 0 (0.0%) | 0 (0.0%) | 0 (0.0%) | |
| CFB ≤ 30 msec | 5 (83.3%) | 6 (100.0%) | 6 (100.0%) | 5 (100.0%) | 6 (100.0%) | 6 (100.0%) | 34 (97.1%) | 12 (100.0%) | |
| CFB > 30 and ≤ 60 msec | 1 (16.7%) | 0 (0.0%) | 0 (0.0%) | 0 (0.0%) | 0 (0.0%) | 0 (0.0%) | 1 (2.9%) | 0 (0.0%) | |
| CFB > 60 msec | 0 (0.0%) | 0 (0.0%) | 0 (0.0%) | 0 (0.0%) | 0 (0.0%) | 0 (0.0%) | 0 (0.0%) | 0 (0.0%) | |
| **0.5 h** | | | | | | | | | |
| QTcF ≤ 450 msec | 6 (100.0%) | 6 (100.0%) | 6 (100.0%) | 5 (100.0%) | 5 (83.3%) | 4 (66.7%) | 32 (91.4%) | 12 (100.0%) | |
| QTcF > 450 msec | 0 (0.0%) | 0 (0.0%) | 0 (0.0%) | 0 (0.0%) | 1 (16.7%) | 1 (16.7%) | 2 (5.7%) | 0 (0.0%) | |
| QTcF > 480 msec | 0 (0.0%) | 0 (0.0%) | 0 (0.0%) | 0 (0.0%) | 0 (0.0%) | 1 (16.7%) | 1 (2.9%) | 0 (0.0%) | |
| QTcF > 500 msec | 0 (0.0%) | 0 (0.0%) | 0 (0.0%) | 0 (0.0%) | 0 (0.0%) | 0 (0.0%) | 0 (0.0%) | 0 (0.0%) | |
| CFB ≤ 30 msec | 6 (100.0%) | 6 (100.0%) | 6 (100.0%) | 4 (80.0%) | 3 (50.0%) | 1 (16.7%) | 26 (74.3%) | 12 (100.0%) | |
| CFB > 30 and ≤ 60 msec | 0 (0.0%) | 0 (0.0%) | 0 (0.0%) | 1 (20.0%) | 3 (50.0%) | 4 (66.7%) | 8 (22.9%) | 0 (0.0%) | |
| CFB > 60 msec | 0 (0.0%) | 0 (0.0%) | 0 (0.0%) | 0 (0.0%) | 0 (0.0%) | 1 (16.7%) | 1 (2.9%) | 0 (0.0%) | |
| **0.75 h** | | | | | | | | | |
| QTcF ≤ 450 msec | 6 (100.0%) | 6 (100.0%) | 6 (100.0%) | 5 (100.0%) | 6 (100.0%) | 5 (83.3%) | 34 (97.1%) | 12 (100.0%) | |
| QTcF > 450 msec | 0 (0.0%) | 0 (0.0%) | 0 (0.0%) | 0 (0.0%) | 0 (0.0%) | 1 (16.7%) | 1 (2.9%) | 0 (0.0%) | |
| QTcF > 480 msec | 0 (0.0%) | 0 (0.0%) | 0 (0.0%) | 0 (0.0%) | 0 (0.0%) | 0 (0.0%) | 0 (0.0%) | 0 (0.0%) | |
| QTcF > 500 msec | 0 (0.0%) | 0 (0.0%) | 0 (0.0%) | 0 (0.0%) | 0 (0.0%) | 0 (0.0%) | 0 (0.0%) | 0 (0.0%) | |
| CFB ≤ 30 msec | 6 (100.0%) | 6 (100.0%) | 6 (100.0%) | 5 (100.0%) | 4 (66.7%) | 3 (50.0%) | 30 (85.7%) | 12 (100.0%) | |
| CFB > 30 and ≤ 60 msec | 0 (0.0%) | 0 (0.0%) | 0 (0.0%) | 0 (0.0%) | 2 (33.3%) | 3 (50.0%) | 5 (14.3%) | 0 (0.0%) | |
| CFB > 60 msec | 0 (0.0%) | 0 (0.0%) | 0 (0.0%) | 0 (0.0%) | 0 (0.0%) | 0 (0.0%) | 0 (0.0%) | 0 (0.0%) | |
| **1 h** | | | | | | | | | |
| QTcF ≤ 450 msec | 6 (100.0%) | 6 (100.0%) | 6 (100.0%) | 5 (100.0%) | 6 (100.0%) | 6 (100.0%) | 35 (100.0%) | 12 (100.0%) | |
| QTcF > 450 msec | 0 (0.0%) | 0 (0.0%) | 0 (0.0%) | 0 (0.0%) | 0 (0.0%) | 0 (0.0%) | 0 (0.0%) | 0 (0.0%) | |
| QTcF > 480 msec | 0 (0.0%) | 0 (0.0%) | 0 (0.0%) | 0 (0.0%) | 0 (0.0%) | 0 (0.0%) | 0 (0.0%) | 0 (0.0%) | |
| QTcF > 500 msec | 0 (0.0%) | 0 (0.0%) | 0 (0.0%) | 0 (0.0%) | 0 (0.0%) | 0 (0.0%) | 0 (0.0%) | 0 (0.0%) | |
| CFB ≤ 30 msec | 6 (100.0%) | 6 (100.0%) | 6 (100.0%) | 5 (100.0%) | 5 (83.3%) | 5 (83.3%) | 33 (94.3%) | 12 (100.0%) | |
| CFB > 30 and ≤ 60 msec | 0 (0.0%) | 0 (0.0%) | 0 (0.0%) | 0 (0.0%) | 1 (16.7%) | 1 (16.7%) | 2 (5.7%) | 0 (0.0%) | |
| CFB > 60 msec | 0 (0.0%) | 0 (0.0%) | 0 (0.0%) | 0 (0.0%) | 0 (0.0%) | 0 (0.0%) | 0 (0.0%) | 0 (0.0%) | |
| **2 h** | | | | | | | | | |
| QTcF ≤ 450 msec | 6 (100.0%) | 6 (100.0%) | 6 (100.0%) | 5 (100.0%) | 6 (100.0%) | 5 (83.3%) | 34 (97.1%) | 12 (100.0%) | |
| QTcF > 450 msec | 0 (0.0%) | 0 (0.0%) | 0 (0.0%) | 0 (0.0%) | 0 (0.0%) | 1 (16.7%) | 1 (2.9%) | 0 (0.0%) | |
| QTcF > 480 msec | 0 (0.0%) | 0 (0.0%) | 0 (0.0%) | 0 (0.0%) | 0 (0.0%) | 0 (0.0%) | 0 (0.0%) | 0 (0.0%) | |
| QTcF > 500 msec | 0 (0.0%) | 0 (0.0%) | 0 (0.0%) | 0 (0.0%) | 0 (0.0%) | 0 (0.0%) | 0 (0.0%) | 0 (0.0%) | |
| CFB ≤ 30 msec | 6 (100.0%) | 6 (100.0%) | 6 (100.0%) | 5 (100.0%) | 6 (100.0%) | 6 (100.0%) | 35 (100.0%) | 12 (100.0%) | |
| CFB > 30 and ≤ 60 msec | 0 (0.0%) | 0 (0.0%) | 0 (0.0%) | 0 (0.0%) | 0 (0.0%) | 0 (0.0%) | 0 (0.0%) | 0 (0.0%) | |
| CFB > 60 msec | 0 (0.0%) | 0 (0.0%) | 0 (0.0%) | 0 (0.0%) | 0 (0.0%) | 0 (0.0%) | 0 (0.0%) | 0 (0.0%) | |
| **3 h** | | | | | | | | | |
| QTcF ≤ 450 msec | 6 (100.0%) | 6 (100.0%) | 6 (100.0%) | 5 (100.0%) | 6 (100.0%) | 5 (83.3%) | 34 (97.1%) | 12 (100.0%) | |
| QTcF > 450 msec | 0 (0.0%) | 0 (0.0%) | 0 (0.0%) | 0 (0.0%) | 0 (0.0%) | 1 (16.7%) | 1 (2.9%) | 0 (0.0%) | |
| QTcF > 480 msec | 0 (0.0%) | 0 (0.0%) | 0 (0.0%) | 0 (0.0%) | 0 (0.0%) | 0 (0.0%) | 0 (0.0%) | 0 (0.0%) | |
| QTcF > 500 msec | 0 (0.0%) | 0 (0.0%) | 0 (0.0%) | 0 (0.0%) | 0 (0.0%) | 0 (0.0%) | 0 (0.0%) | 0 (0.0%) | |
| CFB ≤ 30 msec | 6 (100.0%) | 6 (100.0%) | 6 (100.0%) | 5 (100.0%) | 6 (100.0%) | 5 (83.3%) | 34 (97.1%) | 12 (100.0%) | |
| CFB > 30 and ≤ 60 msec | 0 (0.0%) | 0 (0.0%) | 0 (0.0%) | 0 (0.0%) | 0 (0.0%) | 1 (16.7%) | 1 (2.9%) | 0 (0.0%) | |
| CFB > 60 msec | 0 (0.0%) | 0 (0.0%) | 0 (0.0%) | 0 (0.0%) | 0 (0.0%) | 0 (0.0%) | 0 (0.0%) | 0 (0.0%) | |
| **4 h** | | | | | | | | | |
| QTcF ≤ 450 msec | 6 (100.0%) | 6 (100.0%) | 6 (100.0%) | 5 (100.0%) | 6 (100.0%) | 5 (83.3%) | 34 (97.1%) | 12 (100.0%) | |
| QTcF > 450 msec | 0 (0.0%) | 0 (0.0%) | 0 (0.0%) | 0 (0.0%) | 0 (0.0%) | 1 (16.7%) | 1 (2.9%) | 0 (0.0%) | |
| QTcF > 480 msec | 0 (0.0%) | 0 (0.0%) | 0 (0.0%) | 0 (0.0%) | 0 (0.0%) | 0 (0.0%) | 0 (0.0%) | 0 (0.0%) | |
| QTcF > 500 msec | 0 (0.0%) | 0 (0.0%) | 0 (0.0%) | 0 (0.0%) | 0 (0.0%) | 0 (0.0%) | 0 (0.0%) | 0 (0.0%) | |
| CFB ≤ 30 msec | 6 (100.0%) | 6 (100.0%) | 6 (100.0%) | 5 (100.0%) | 6 (100.0%) | 6 (100.0%) | 35 (100.0%) | 12 (100.0%) | |
| CFB > 30 and ≤ 60 msec | 0 (0.0%) | 0 (0.0%) | 0 (0.0%) | 0 (0.0%) | 0 (0.0%) | 0 (0.0%) | 0 (0.0%) | 0 (0.0%) | |
| CFB > 60 msec | 0 (0.0%) | 0 (0.0%) | 0 (0.0%) | 0 (0.0%) | 0 (0.0%) | 0 (0.0%) | 0 (0.0%) | 0 (0.0%) | |
| **6 h** | | | | | | | | | |
| QTcF ≤ 450 msec | 6 (100.0%) | 6 (100.0%) | 6 (100.0%) | 5 (100.0%) | 6 (100.0%) | 6 (100.0%) | 35 (100.0%) | 12 (100.0%) | |
| QTcF > 450 msec | 0 (0.0%) | 0 (0.0%) | 0 (0.0%) | 0 (0.0%) | 0 (0.0%) | 0 (0.0%) | 0 (0.0%) | 0 (0.0%) | |
| QTcF > 480 msec | 0 (0.0%) | 0 (0.0%) | 0 (0.0%) | 0 (0.0%) | 0 (0.0%) | 0 (0.0%) | 0 (0.0%) | 0 (0.0%) | |
| QTcF > 500 msec | 0 (0.0%) | 0 (0.0%) | 0 (0.0%) | 0 (0.0%) | 0 (0.0%) | 0 (0.0%) | 0 (0.0%) | 0 (0.0%) | |
| CFB ≤ 30 msec | 6 (100.0%) | 6 (100.0%) | 6 (100.0%) | 5 (100.0%) | 6 (100.0%) | 6 (100.0%) | 35 (100.0%) | 12 (100.0%) | |
| CFB > 30 and ≤ 60 msec | 0 (0.0%) | 0 (0.0%) | 0 (0.0%) | 0 (0.0%) | 0 (0.0%) | 0 (0.0%) | 0 (0.0%) | 0 (0.0%) | |
| CFB > 60 msec | 0 (0.0%) | 0 (0.0%) | 0 (0.0%) | 0 (0.0%) | 0 (0.0%) | 0 (0.0%) | 0 (0.0%) | 0 (0.0%) | |
| **8 h** | | | | | | | | | |
| QTcF ≤ 450 msec | 6 (100.0%) | 6 (100.0%) | 6 (100.0%) | 5 (100.0%) | 6 (100.0%) | 6 (100.0%) | 35 (100.0%) | 12 (100.0%) | |
| QTcF > 450 msec | 0 (0.0%) | 0 (0.0%) | 0 (0.0%) | 0 (0.0%) | 0 (0.0%) | 0 (0.0%) | 0 (0.0%) | 0 (0.0%) | |
| QTcF > 480 msec | 0 (0.0%) | 0 (0.0%) | 0 (0.0%) | 0 (0.0%) | 0 (0.0%) | 0 (0.0%) | 0 (0.0%) | 0 (0.0%) | |
| QTcF > 500 msec | 0 (0.0%) | 0 (0.0%) | 0 (0.0%) | 0 (0.0%) | 0 (0.0%) | 0 (0.0%) | 0 (0.0%) | 0 (0.0%) | |
| CFB ≤ 30 msec | 6 (100.0%) | 6 (100.0%) | 6 (100.0%) | 5 (100.0%) | 6 (100.0%) | 6 (100.0%) | 35 (100.0%) | 12 (100.0%) | |
| CFB > 30 and ≤ 60 msec | 0 (0.0%) | 0 (0.0%) | 0 (0.0%) | 0 (0.0%) | 0 (0.0%) | 0 (0.0%) | 0 (0.0%) | 0 (0.0%) | |
| CFB > 60 msec | 0 (0.0%) | 0 (0.0%) | 0 (0.0%) | 0 (0.0%) | 0 (0.0%) | 0 (0.0%) | 0 (0.0%) | 0 (0.0%) | |
| **12 h** | | | | | | | | | |
| QTcF ≤ 450 msec | 6 (100.0%) | 6 (100.0%) | 6 (100.0%) | 5 (100.0%) | 6 (100.0%) | 6 (100.0%) | 35 (100.0%) | 12 (100.0%) | |
| QTcF > 450 msec | 0 (0.0%) | 0 (0.0%) | 0 (0.0%) | 0 (0.0%) | 0 (0.0%) | 0 (0.0%) | 0 (0.0%) | 0 (0.0%) | |
| QTcF > 480 msec | 0 (0.0%) | 0 (0.0%) | 0 (0.0%) | 0 (0.0%) | 0 (0.0%) | 0 (0.0%) | 0 (0.0%) | 0 (0.0%) | |
| QTcF > 500 msec | 0 (0.0%) | 0 (0.0%) | 0 (0.0%) | 0 (0.0%) | 0 (0.0%) | 0 (0.0%) | 0 (0.0%) | 0 (0.0%) | |
| CFB ≤ 30 msec | 6 (100.0%) | 6 (100.0%) | 6 (100.0%) | 5 (100.0%) | 6 (100.0%) | 6 (100.0%) | 35 (100.0%) | 12 (100.0%) | |
| CFB > 30 and ≤ 60 msec | 0 (0.0%) | 0 (0.0%) | 0 (0.0%) | 0 (0.0%) | 0 (0.0%) | 0 (0.0%) | 0 (0.0%) | 0 (0.0%) | |
| CFB > 60 msec | 0 (0.0%) | 0 (0.0%) | 0 (0.0%) | 0 (0.0%) | 0 (0.0%) | 0 (0.0%) | 0 (0.0%) | 0 (0.0%) | |
| **24 h** | | | | | | | | | |
| QTcF ≤ 450 msec | 6 (100.0%) | 6 (100.0%) | 6 (100.0%) | 5 (100.0%) | 6 (100.0%) | 6 (100.0%) | 35 (100.0%) | 11 (100.0%) | |
| QTcF > 450 msec | 0 (0.0%) | 0 (0.0%) | 0 (0.0%) | 0 (0.0%) | 0 (0.0%) | 0 (0.0%) | 0 (0.0%) | 0 (0.0%) | |
| QTcF > 480 msec | 0 (0.0%) | 0 (0.0%) | 0 (0.0%) | 0 (0.0%) | 0 (0.0%) | 0 (0.0%) | 0 (0.0%) | 0 (0.0%) | |
| QTcF > 500 msec | 0 (0.0%) | 0 (0.0%) | 0 (0.0%) | 0 (0.0%) | 0 (0.0%) | 0 (0.0%) | 0 (0.0%) | 0 (0.0%) | |
| CFB ≤ 30 msec | 6 (100.0%) | 6 (100.0%) | 6 (100.0%) | 5 (100.0%) | 6 (100.0%) | 6 (100.0%) | 35 (100.0%) | 11 (100.0%) | |
| CFB > 30 and ≤ 60 msec | 0 (0.0%) | 0 (0.0%) | 0 (0.0%) | 0 (0.0%) | 0 (0.0%) | 0 (0.0%) | 0 (0.0%) | 0 (0.0%) | |
| CFB > 60 msec | 0 (0.0%) | 0 (0.0%) | 0 (0.0%) | 0 (0.0%) | 0 (0.0%) | 0 (0.0%) | 0 (0.0%) | 0 (0.0%) | |
| CFB = change from baseline; QTcF = Fridericia-corrected QT interval  Note: N is the number of subjects in the ECG Population in the specified category, at the corresponding time point. Percent (%) is the percentage of the subjects in the ECG Population in the specified category at the time point. Any instance in one, two or three of the triplicate ECGs where the criteria are met at each time point is counted.  Program: tholtfrq.sas (run date: 24SEP2018) Data Source: ADEGPC | | | | | | | | |  |

**Table A2: Categorical Summaries of Post-Baseline HR Outliers (ECG Population)**

| **HR Interval**  **Time Point** | **Cohort A**  **20 mg** | **Cohort B**  **60 mg** | **Cohort C**  **180 mg** | **Cohort D**  **360 mg** | **Cohort E**  **600 mg** | **Cohort F**  **600 mg** | **Combined**  **HBI-3000** | **Placebo** |
| --- | --- | --- | --- | --- | --- | --- | --- | --- |
| **HR < 50 bpm and a 25% or greater decrease from baseline** | | | | | | | | |
| Minimum value post dose | 0/6 (0.0%) | 0/6 (0.0%) | 0/6 (0.0%) | 0/5 (0.0%) | 0/6 (0.0%) | 0/6 (0.0%) | 0/35 (0.0%) | 0/12 (0.0%) |
| 0.25 h | 0/6 (0.0%) | 0/6 (0.0%) | 0/6 (0.0%) | 0/5 (0.0%) | 0/6 (0.0%) | 0/6 (0.0%) | 0/35 (0.0%) | 0/12 (0.0%) |
| 0.5 h | 0/6 (0.0%) | 0/6 (0.0%) | 0/6 (0.0%) | 0/5 (0.0%) | 0/6 (0.0%) | 0/6 (0.0%) | 0/35 (0.0%) | 0/12 (0.0%) |
| 0.75 h | 0/6 (0.0%) | 0/6 (0.0%) | 0/6 (0.0%) | 0/5 (0.0%) | 0/6 (0.0%) | 0/6 (0.0%) | 0/35 (0.0%) | 0/12 (0.0%) |
| 1 h | 0/6 (0.0%) | 0/6 (0.0%) | 0/6 (0.0%) | 0/5 (0.0%) | 0/6 (0.0%) | 0/6 (0.0%) | 0/35 (0.0%) | 0/12 (0.0%) |
| 2 h | 0/6 (0.0%) | 0/6 (0.0%) | 0/6 (0.0%) | 0/5 (0.0%) | 0/6 (0.0%) | 0/6 (0.0%) | 0/35 (0.0%) | 0/12 (0.0%) |
| 3 h | 0/6 (0.0%) | 0/6 (0.0%) | 0/6 (0.0%) | 0/5 (0.0%) | 0/6 (0.0%) | 0/6 (0.0%) | 0/35 (0.0%) | 0/12 (0.0%) |
| 4 h | 0/6 (0.0%) | 0/6 (0.0%) | 0/6 (0.0%) | 0/5 (0.0%) | 0/6 (0.0%) | 0/6 (0.0%) | 0/35 (0.0%) | 0/12 (0.0%) |
| 6 h | 0/6 (0.0%) | 0/6 (0.0%) | 0/6 (0.0%) | 0/5 (0.0%) | 0/6 (0.0%) | 0/6 (0.0%) | 0/35 (0.0%) | 0/12 (0.0%) |
| 8 h | 0/6 (0.0%) | 0/6 (0.0%) | 0/6 (0.0%) | 0/5 (0.0%) | 0/6 (0.0%) | 0/6 (0.0%) | 0/35 (0.0%) | 0/12 (0.0%) |
| 12 h | 0/6 (0.0%) | 0/6 (0.0%) | 0/6 (0.0%) | 0/5 (0.0%) | 0/6 (0.0%) | 0/6 (0.0%) | 0/35 (0.0%) | 0/12 (0.0%) |
| 24 h | 0/6 (0.0%) | 0/6 (0.0%) | 0/6 (0.0%) | 0/5 (0.0%) | 0/6 (0.0%) | 0/6 (0.0%) | 0/35 (0.0%) | 0/11 (0.0%) |
| **HR > 100 bpm and a 25% or greater increase from baseline** | | | | | | | | |
| Maximum value post dose | 0/6 (0.0%) | 0/6 (0.0%) | 0/6 (0.0%) | 0/5 (0.0%) | 0/6 (0.0%) | 1/6 (16.7%) | 1/35 (2.9%) | 0/12 (0.0%) |
| 0.25 h | 0/6 (0.0%) | 0/6 (0.0%) | 0/6 (0.0%) | 0/5 (0.0%) | 0/6 (0.0%) | 0/6 (0.0%) | 0/35 (0.0%) | 0/12 (0.0%) |
| 0.5 h | 0/6 (0.0%) | 0/6 (0.0%) | 0/6 (0.0%) | 0/5 (0.0%) | 0/6 (0.0%) | 0/6 (0.0%) | 0/35 (0.0%) | 0/12 (0.0%) |
| 0.75 h | 0/6 (0.0%) | 0/6 (0.0%) | 0/6 (0.0%) | 0/5 (0.0%) | 0/6 (0.0%) | 1/6 (16.7%) | 1/35 (2.9%) | 0/12 (0.0%) |
| 1 h | 0/6 (0.0%) | 0/6 (0.0%) | 0/6 (0.0%) | 0/5 (0.0%) | 0/6 (0.0%) | 0/6 (0.0%) | 0/35 (0.0%) | 0/12 (0.0%) |
| 2 h | 0/6 (0.0%) | 0/6 (0.0%) | 0/6 (0.0%) | 0/5 (0.0%) | 0/6 (0.0%) | 0/6 (0.0%) | 0/35 (0.0%) | 0/12 (0.0%) |
| 3 h | 0/6 (0.0%) | 0/6 (0.0%) | 0/6 (0.0%) | 0/5 (0.0%) | 0/6 (0.0%) | 0/6 (0.0%) | 0/35 (0.0%) | 0/12 (0.0%) |
| 4 h | 0/6 (0.0%) | 0/6 (0.0%) | 0/6 (0.0%) | 0/5 (0.0%) | 0/6 (0.0%) | 0/6 (0.0%) | 0/35 (0.0%) | 0/12 (0.0%) |
| 6 h | 0/6 (0.0%) | 0/6 (0.0%) | 0/6 (0.0%) | 0/5 (0.0%) | 0/6 (0.0%) | 0/6 (0.0%) | 0/35 (0.0%) | 0/12 (0.0%) |
| 8 h | 0/6 (0.0%) | 0/6 (0.0%) | 0/6 (0.0%) | 0/5 (0.0%) | 0/6 (0.0%) | 0/6 (0.0%) | 0/35 (0.0%) | 0/12 (0.0%) |
| 12 h | 0/6 (0.0%) | 0/6 (0.0%) | 0/6 (0.0%) | 0/5 (0.0%) | 0/6 (0.0%) | 0/6 (0.0%) | 0/35 (0.0%) | 0/12 (0.0%) |
| 24 h | 0/6 (0.0%) | 0/6 (0.0%) | 0/6 (0.0%) | 0/5 (0.0%) | 0/6 (0.0%) | 0/6 (0.0%) | 0/35 (0.0%) | 0/11 (0.0%) |
| Note: N is the number of subjects in the ECG Population in the specified category, at the corresponding time point. Percent (%) is the percentage of the subjects in the ECG Population in the specified category at the time point. Any instance in one, two, or three of the triplicate ECGs where the criteria are met at each time point is counted as one outlier event.  Program: tholtout.sas (Run Date: 24SEP2018) Data Source: ADEGPC | | | | | | | | |

**Table A3: Categorical Summaries of Post-Baseline PR Interval Outliers (ECG Population)**

| **PR Interval**  **Time Point** | **Cohort A**  **20 mg** | **Cohort B**  **60 mg** | **Cohort C**  **180 mg** | **Cohort D**  **360 mg** | **Cohort E**  **600 mg** | **Cohort F**  **600 mg** | **Combined**  **HBI-3000** | **Placebo** |
| --- | --- | --- | --- | --- | --- | --- | --- | --- |
| **PR > 200 msec and a 25% or greater increase from baseline** | | | | | | | | |
| Maximum value post dose | 0/6 (0.0%) | 1/6 (16.7%) | 0/6 (0.0%) | 1/5 (20.0%) | 0/6 (0.0%) | 2/6 (33.3%) | 4/35 (11.4%) | 0/12 (0.0%) |
| 0.25 h | 0/6 (0.0%) | 0/6 (0.0%) | 0/6 (0.0%) | 0/5 (0.0%) | 0/6 (0.0%) | 0/6 (0.0%) | 0/35 (0.0%) | 0/12 (0.0%) |
| 0.5 h | 0/6 (0.0%) | 0/6 (0.0%) | 0/6 (0.0%) | 0/5 (0.0%) | 0/6 (0.0%) | 2/6 (33.3%) | 2/35 (5.7%) | 0/12 (0.0%) |
| 0.75 h | 0/6 (0.0%) | 0/6 (0.0%) | 0/6 (0.0%) | 1/5 (20.0%) | 0/6 (0.0%) | 0/6 (0.0%) | 1/35 (2.9%) | 0/12 (0.0%) |
| 1 h | 0/6 (0.0%) | 1/6 (16.7%) | 0/6 (0.0%) | 0/5 (0.0%) | 0/6 (0.0%) | 0/6 (0.0%) | 1/35 (2.9%) | 0/12 (0.0%) |
| 2 h | 0/6 (0.0%) | 0/6 (0.0%) | 0/6 (0.0%) | 0/5 (0.0%) | 0/6 (0.0%) | 0/6 (0.0%) | 0/35 (0.0%) | 0/12 (0.0%) |
| 3 h | 0/6 (0.0%) | 0/6 (0.0%) | 0/6 (0.0%) | 0/5 (0.0%) | 0/6 (0.0%) | 0/6 (0.0%) | 0/35 (0.0%) | 0/12 (0.0%) |
| 4 h | 0/6 (0.0%) | 0/6 (0.0%) | 0/6 (0.0%) | 0/5 (0.0%) | 0/6 (0.0%) | 0/6 (0.0%) | 0/35 (0.0%) | 0/12 (0.0%) |
| 6 h | 0/6 (0.0%) | 0/6 (0.0%) | 0/6 (0.0%) | 0/5 (0.0%) | 0/6 (0.0%) | 0/6 (0.0%) | 0/35 (0.0%) | 0/12 (0.0%) |
| 8 h | 0/6 (0.0%) | 0/6 (0.0%) | 0/6 (0.0%) | 0/5 (0.0%) | 0/6 (0.0%) | 0/6 (0.0%) | 0/35 (0.0%) | 0/12 (0.0%) |
| 12 h | 0/6 (0.0%) | 0/6 (0.0%) | 0/6 (0.0%) | 0/5 (0.0%) | 0/6 (0.0%) | 0/6 (0.0%) | 0/35 (0.0%) | 0/12 (0.0%) |
| 24 h | 0/6 (0.0%) | 0/6 (0.0%) | 0/6 (0.0%) | 0/5 (0.0%) | 0/6 (0.0%) | 0/6 (0.0%) | 0/35 (0.0%) | 0/11 (0.0%) |
| Note: N is the number of subjects in the ECG Population in the specified category, at the corresponding time point. Percent (%) is the percentage of the subjects in the ECG Population in the specified category at the time point. Any instance in one, two, or three of the triplicate ECGs where the criteria are met at each time point is counted as one outlier event.  Program: tholtout.sas (Run Date: 24SEP2018) Data Source: ADEGPC | | | | | | | | |

**ΔECG Concentration-Effect Plots**

**Figure A1: ΔQTcF**

**Figure A2: ΔHR**

**Figure A3: ΔPR**

**Figure A4: ΔP_Dur_**

**Figure A5: ΔQRS**

**Figure A6: ΔJTpc**

**Figure A7: ΔTpTe**

**Supplement**

**Section B**

**Assessment of the Effect of Denoising on ECG Results**

**ECG Selection**

ECGs were selected for evaluation of the effect on denoising on interval measurements in two steps. The first step was to identify three timepoints that were clear of interference, as judged by a tally done by BioMedical Systems, in all nine subjects whose ECG recordings had been affected by AC interference. It was not possible to use the same time points across all subjects. Baseline ECGs were also selected for each of those nine subjects as the pre-dose time point on Day 1. At each 5-minute time point window, triplicate ECGs, 1 minute apart in time, were analyzed without averaging.

The second step was to examine the ECGs at each of the purportedly non-noisy time points and exclude any subjects/time points that were in fact affected by prominent AC interference and were therefore not clean. The unfiltered, presumably non-noisy ECGs intended for this analysis were originally thought to be free of 50 Hz interference, as they had not been flagged as unreadable by the ECG Core Laboratory. However, upon inspection we found that unfiltered ECGs from 12 of the 36 total filtered/unfiltered ECG pairs were sufficiently affected by AC interference to be unmeasurable, resulting in 24 time points (72 ECG pairs) that required denoising analysis of ECG intervals to be successfully measured. Two of the unmeasurable sets were from the baseline time point, which meant that all of their accompanying post-baseline time points had to be excluded from the ΔECG (change from baseline) analysis, resulting in 19 analyzable time points (57 ECG pairs).

**Statistical Methods**

Filtered and unfiltered ECG and derived ECG intervals from the pre-specified time points were compared using summary statistics and linear regression for the whole population combined and by time point and individual subject. All statistical analyses were done using JMP v13 (SAS Institute, Cary, NC).

**Results**

**By Time Point**

**ECG**

In Table B1, all of the aforementioned nine subjects and their analyzable time points were combined to provide a population-wide assessment of the differences between filtered and unfiltered ECG interval measurements. The mean population differences as a proportion of the mean unfiltered values are generally small (-1.4% to 0.9%) for all but the QRS interval, which had a 4.3% variation. This low variation between filtered and unfiltered ECG population average ECG intervals suggests that the filtered data can be relied upon for population estimates both in an entirely filtered dataset and in one combining filtered with unfiltered data, with the exception of the filtered QRS interval. A more definitive assessment of this conclusion presented in section 3.1.1.2 was based on the differences seen for ΔECG. In the main report, only filtered data were analyzed.

As expected, the absolute differences between filtered and unfiltered measurements are larger than the mean differences, but the maximum absolute differences were small (less than 1.8 msec), with the exception of the QRS interval (4.4%). This variation is probably small enough to allow filtered data (with the exception of the QRS interval) to be reliably used to tally categorical or outlier changes in the population. In the main report, only filtered data were analyzed.

**Table B1: Filtered Minus Unfiltered ECG, Mean and Absolute Mean, msec**

| **Variable** | **N** | **Mean** | **Std Dev** | **Lower 95% Mean** | **Upper 95% Mean** | **Unfiltered ECG Mean** | **% of Unfiltered ECG Mean** |
| --- | --- | --- | --- | --- | --- | --- | --- |
| RR difference | 72 | 1.4 | 4.00 | 0.4 | 2.3 | 925.4 | 0.2 |
| PR difference | 72 | -2.3 | 1.60 | -2.7 | -1.9 | 165.3 | -1.4 |
| QRS difference | 72 | 4.2 | 1.67 | 3.8 | 4.6 | 97.9 | 4.3 |
| QT difference | 72 | 3.5 | 2.23 | 3.0 | 4.1 | 394.2 | 0.9 |
| JTp difference | 72 | -1.9 | 1.16 | -2.1 | -1.6 | 206.7 | -0.9 |
| TpTe difference | 72 | 1.2 | 2.51 | 0.6 | 1.8 | 89.6 | 1.3 |
| Absolute RR difference | 72 | 3.6 | 2.17 | 3.1 | 4.1 | 925.4 | 0.4 |
| Absolute PR difference | 72 | 2.4 | 1.33 | 2.1 | 2.6 | 165.3 | 1.5 |
| Absolute QRS difference | 72 | 4.3 | 1.52 | 3.9 | 4.6 | 97.9 | 4.4 |
| Absolute QT difference | 72 | 3.6 | 2.14 | 3.1 | 4.1 | 394.2 | 0.9 |
| Absolute JTP difference | 72 | 1.9 | 1.16 | 1.6 | 2.1 | 206.7 | 0.9 |
| Absolute TpTe difference | 72 | 1.5 | 2.33 | 1.0 | 2.1 | 89.6 | 1.7 |
| N = The number of ECGs contributing to the analysis (24 time points, three ECGs per time point) | | | | | | | |

**ΔECG**

In Table B2, all of the nine subjects and their corresponding time points were combined to provide a population-wide assessment of the differences between filtered and unfiltered ΔECG (change from baseline) interval measurements. Both the mean population ΔECG difference values and those calculated as a proportion of the unfiltered mean ECG values were considerably smaller (-0.4% to 0.2%) than those of ECG difference, an outcome that would be expected if the distortion caused by filtering was systematic, i.e., affecting baseline and on treatment values equally. The small mean population differences suggest that the filtered data can be relied upon for making population estimates of ΔECG both in an entirely filtered dataset and in one that combines filtered and unfiltered data, with no exceptions.

As expected, the absolute differences between filtered and unfiltered measurements were larger. However, the largest proportional difference was only 1.1%, a small enough variation that indicates filtered data can be used reliably to tally categorical or outlier changes of ΔECG in the population.

**Table B2: Filtered Minus Unfiltered ΔECG, Mean and Absolute Mean, msec**

| **Variable** | **N** | **Mean** | **Std Dev** | **Lower 95% Mean** | **Upper 95% Mean** | **Unfiltered ECG Mean** | **% Unfiltered  ECG Mean** |
| --- | --- | --- | --- | --- | --- | --- | --- |
| dΔRR difference | 57 | 0.4 | 4.53 | -0.8 | 1.6 | 925.4 | 0.0 |
| ΔPR difference | 57 | 0.3 | 1.18 | -0.0 | 0.6 | 165.3 | 0.2 |
| ΔQRS difference | 57 | -0.4 | 1.50 | -0.8 | -0.0 | 97.9 | -0.4 |
| ΔQT difference | 57 | -0.1 | 1.53 | -0.5 | 0.3 | 394.2 | -0.0 |
| ΔJTp difference | 57 | 0.1 | 0.91 | -0.2 | 0.3 | 206.7 | 0.0 |
| ΔTpTe difference | 57 | 0.2 | 1.14 | -0.1 | 0.5 | 89.6 | 0.2 |
| Absolute ΔRR difference | 57 | 3.2 | 3.25 | 2.3 | 4.0 | 925.4 | 0.3 |
| Absolute ΔPR difference | 57 | 0.8 | 0.90 | 0.6 | 1.0 | 165.3 | 0.5 |
| Absolute ΔQRS difference | 57 | 1.1 | 1.14 | 0.8 | 1.4 | 97.9 | 1.1 |
| Absolute ΔQT difference | 57 | 1.0 | 1.17 | 0.7 | 1.3 | 394.2 | 0.3 |
| Absolute ΔJTp difference | 57 | 0.6 | 0.71 | 0.4 | 0.8 | 206.7 | 0.3 |
| Absolute ΔTpTe difference | 57 | 0.8 | 0.87 | 0.5 | 1.0 | 89.6 | 0.9 |
| N = The number of ECGs contributing to the analysis (19 time points, three ECGs per time point) | | | | | | | |

**Linear Regressions**

Linear regressions were constructed with the filtered results as the dependent variable and the unfiltered data the independent variable. Regressions that also included subject as a random effect yielded similar results and are not displayed in this section.

**ECG**

Figure B1 displays linear regressions of filtered on unfiltered data for the four ECG intervals. The fit parameters are displayed in Table B3. A red line in each graph represents the line of identity and the green line the linear fit. The line of identity is overlain by the fit line and cannot be seen in the RR plot. There are small-to-systematic differences between the filtered and unfiltered data for the other parameters, but, as indicated in Table B3, filtered and unfiltered values are highly correlated.

**Figure B1: Linear Regression of Filtered on Unfiltered ECG Values**

RR Interval PR Interval

QRS interval QT Interval

**Table B3: Linear Regression Fit Parameters, ECG**

| **ECG Parameter** | **Intercept Parameter Estimate, msec** | **Unfiltered Parameter Estimate, msec** | ***P* Value** | **R^2^** |
| --- | --- | --- | --- | --- |
| RR | -2.907 | 1.004 | < .0001 | 0.999 |
| PR | -1.429 | 0.995 | < .0001 | 0.974 |
| QRS | 13.205 | 0.908 | < .0001 | 0.963 |
| QT | 2.621 | 1.002 | < .0001 | 0.996 |

**ΔECG**

The discrepancies between the line of fit and the linear relationship between filtered and unfiltered values are considerably diminished for ΔECG measurements (Figure B2 and Table B4). As explained above (section 3.1.1.2), this outcome suggests that the filtering effect was systematically present and consistent at baseline and during treatment, allowing baseline subtraction to reduce the small bias documented in sections 3.1.1.1 and 3.1.2.1.

**Figure B2: Linear Regression of Filtered on Unfiltered ΔECG Values**

RR Interval PR Interval

QRS Interval QT Interval

**Table B4: Linear Regression Fit Parameters, ΔECG**

| **ECG Parameter** | **Intercept Parameter Estimate, msec** | **Unfiltered Parameter Estimate, msec** | ***P* Value** | **R^2^** |
| --- | --- | --- | --- | --- |
| ΔRR | 0.331 | 0.996 | < .0001 | 0.998 |
| ΔPR | 0.157 | 0.918 | < .0001 | 0.908 |
| ΔQRS | .012 | 0.686 | < .0001 | 0.725 |
| ΔQT | -0.061 | 0.983 | < .0001 | 0.987 |

**Comparisons by Time Point by Subject**

Trellis plots showing filtered minus unfiltered ECG and ΔECG values by subject and time point appear in Figure B3 and B4, respectively. The differences between filtered and unfiltered individual ECG values are small, ranging from -2 msec to 7 msec for RR, -5 msec to 4 msec for PR, 2 msec to 6 msec for QRS and 2 msec to 6 msec for QT. Similar results were seen for ΔECG values, ranging from -2 msec to 7 msec for RR, -1 msec to 2 msec for PR, -1 msec to 2 msec for QRS, and -1 msec to 2 msec for QT.

**Figure B3: Filtered Minus Unfiltered ECG and Absolute ECG Values by Subject and Time Point**

RR Interval PR Interval

QRS Interval QT interval

**Figure B4: Filtered Minus Unfiltered** Δ**ECG and Absolute** Δ**ECG Values by Subject and Time Point**

RR Interval PR Interval

QRS Interval QT Interval

**Summary and Conclusions**

The primary finding of this analysis was that notch and bandpass filtration of 50 Hz interference-affected ECGs resulted in measurable differences between the filtered and unfiltered signals, but that these differences were quite small for all but the QRS interval. Though the changes were systematic (PR decreased, QRS and QT increased, and RR unchanged), the population mean changes were small enough for RR, PR, and QT that they could be used reliably in population-level analyses as replacement for or in combination with unfiltered data, so long as each subject’s analyzed data were either all filtered or all unfiltered. The baseline-subtracted differences (ΔECG) were small for all intervals including the QRS, such that all four filtered intervals could be used reliably in population-level analyses.

At the subject/time point level, the distortion caused by filtering was also small; no individual ΔECG difference exceeded 2.0% of the average interval value (RR 0.8%, PR 1.2%, QRS 2.5%, and QT 0.5%). Thus, filtered data can be used reliably to detect outliers or to categorize ECG data as replacement for, or in combination with unfiltered data (though only filtered data were analyzed in the main report).

**Supplement**

**Section C**

**Analysis of Blood Pressure Findings**

**Systolic Blood Pressure**

As shown in Tables C1 and C2, the early change in systolic blood pressure from the immediate pre-drug baseline level during the first 4 hours was variable at the 20 mg dose, but was more consistently negative for the 60, 180, 360, and 600 mg doses of HBI-3000. A dose-response pattern was present, primarily at the 15-minute and 30-minute time points. Systolic BP was also modestly decreased in the placebo group throughout the 4-hour observation period. The largest mean fall in SBP in the first 4 hours, 7.1 mmHg, was observed at 30 minutes, at the conclusion of the infusion of the highest dose of 600 mg HBI-3000 (and maximum sulcardine plasma concentration), at which time the reduction in the placebo group was 3.5 mmHg.

**Table C1: Systolic Blood Pressure Change from Baseline ± SD, mmHg**

| **Hours** | **Placebo** | **20 mg** | **60 mg** | **180 mg** | **360 mg** | **600 mg** |
| --- | --- | --- | --- | --- | --- | --- |
| 0.25 | -2.9 ± 5.55 | -1.0 ± 3.85 | -3.5 ± 4.09 | -3.7 ± 4.37 | -4.4 ± 2.88 | -5.2 ± 6.90 |
| 0.5 | -3.5 ± 6.52 | 2.8 ± 6.85 | -2.7 ± 6.77 | -4.0 ± 5.33 | -5.4 ± 7.92 | -7.1 ± 5.48 |
| 1 | -0.7 ± 8.95 | -2.3 ± 5.75 | -2.7 ± 5.13 | 1.8 ± 6.91 | -1.8 ± 7.95 | -0.7 ± 7.46 |
| 2 | -1.1 ± 10.31 | -1.5 ± 4.59 | -6.3 ± 6.92 | 2.2 ± 9.02 | -5.2 ± 4.15 | -4.2 ± 6.39 |
| 4 | -3.8 ± 5.51 | -5.0 ± 8.74 | -3.3 ± 10.82 | -0.8 ± 3.37 | -3.0 ± 4.74 | -2.4 ± 4.12 |

**Figure C1: Systolic Blood Pressure Change from Baseline ± SD, mmHg**


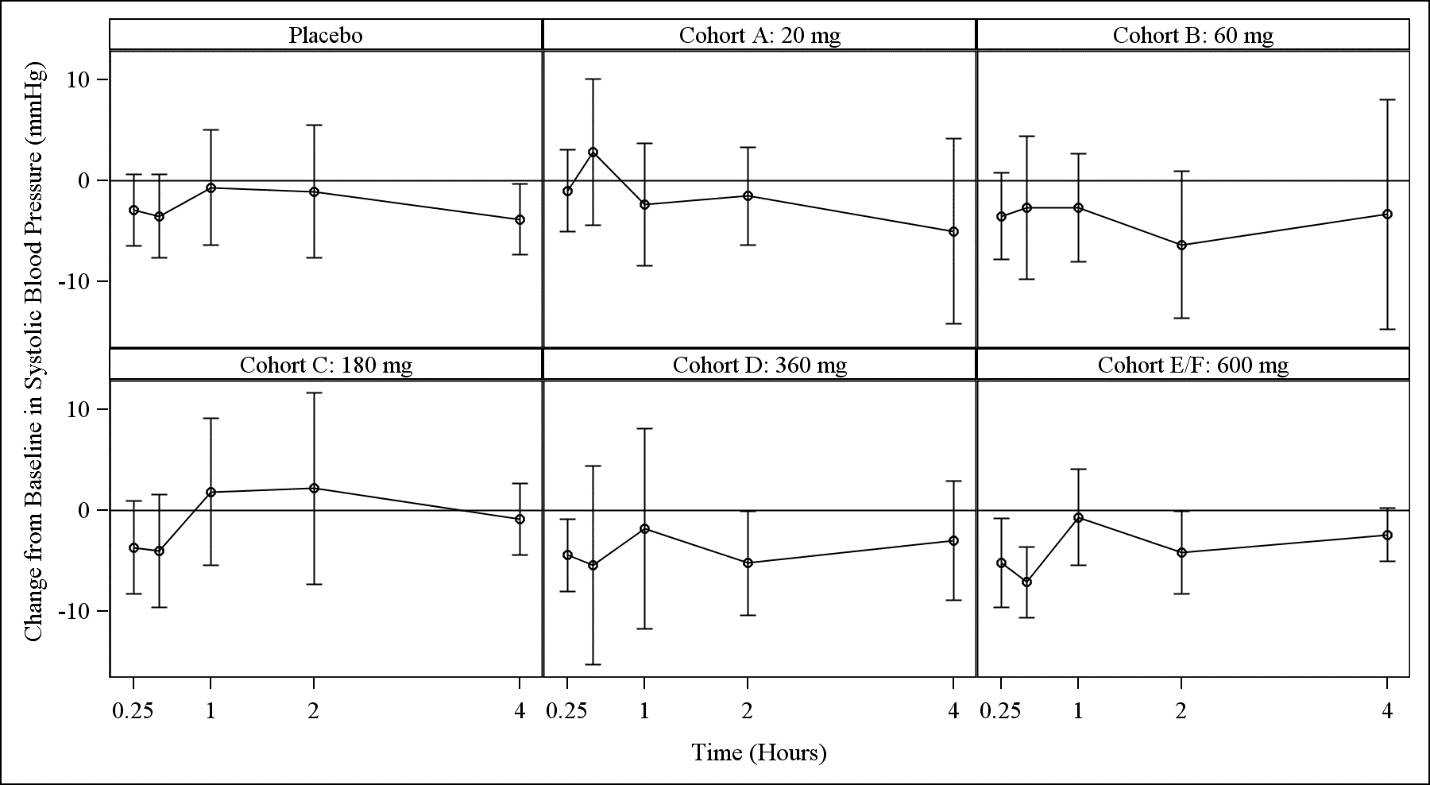


**Diastolic Blood Pressure**

As shown in Table C2 and Figure C3, the early change in diastolic blood pressure from the immediate pre-drug baseline level was generally positive, though a dose-response pattern was not discernible. The largest mean increase in diastolic BP in the first 4 hours, 4.8 mmHg, was observed at 30 minutes at the highest dose of 600 mg, at which time the reduction in the placebo group was 0.3 mmHg.

**Table C2: Diastolic Blood Pressure Change from Baseline ± SD, mmHg**

| **Hours** | **Placebo** | **20 mg** | **60 mg** | **180 mg** | **360 mg** | **600 mg** |
| --- | --- | --- | --- | --- | --- | --- |
| 0.25 | -0.6 ± 5.52 | 3.0 ± 3.41 | 0.0 ± 4.69 | 1.5 ± 4.59 | 1.6 ± 6.84 | 1.6 ± 7.05 |
| 0.5 | -0.3 ± 4.81 | 3.8 ± 8.08 | 1.8 ± 7.31 | 3.8 ± 4.62 | -0.4 ± 7.70 | 4.8 ± 6.03 |
| 1 | -1.7 ± 4.42 | 2.7 ± 6.02 | 0.2 ± 6.49 | 1.0 ± 5.44 | 0.0 ± 8.03 | 4.7 ± 5.57 |
| 2 | -2.2 ± 6.21 | 3.5 ± 5.21 | -3.0 ± 6.87 | -1.5 ± 2.26 | -1.0 ± 5.00 | 0.2 ± 5.13 |
| 4 | -2.2 ± 6.38 | -1.7 ± 6.83 | -2.8 ± 7.55 | -1.2 ± 6.91 | -1.6 ± 8.82 | 1.7 ± 4.70 |

**Figure C2: Diastolic Blood Pressure Change from Baseline ± SD, mmHg**


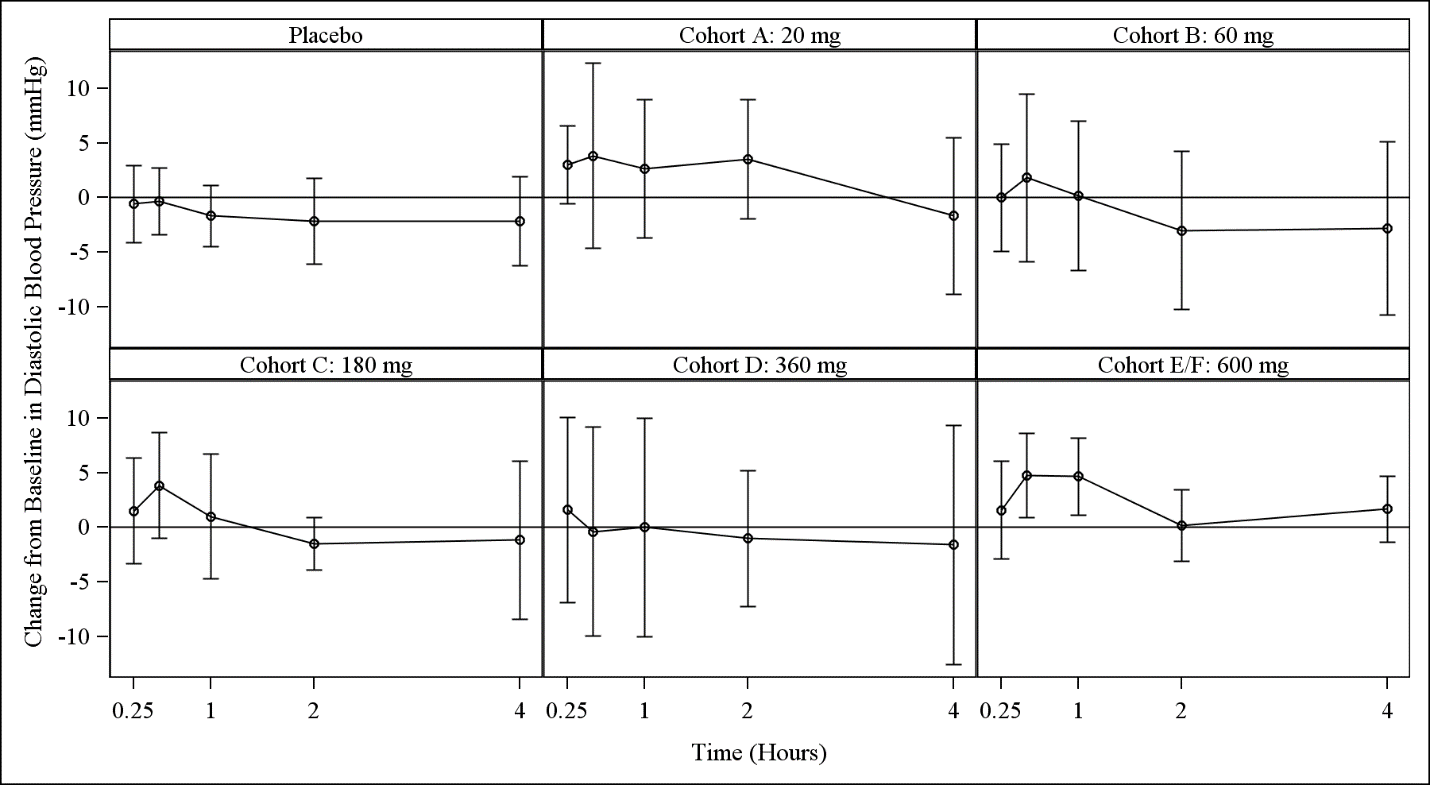


**Mean Blood Pressure**

Mean blood pressure was calculated as (systolic BP + 2 x diastolic BP) ÷ 3. As shown in Table C3 and Figure C3, the early change in mean blood pressure from the immediate pre-drug baseline level was considerably smaller than the systolic and diastolic changes, and a dose-response pattern was absent. At the highest dose of 600 mg (at 30 minutes), mean BP was slightly above zero.

**Table C3: Mean Blood Pressure Change from Baseline ± SD, mmHg**

| **Minutes** | **Placebo** | **20 mg** | **60 mg** | **180 mg** | **360 mg** | **600 mg** |
| --- | --- | --- | --- | --- | --- | --- |
| 15 | -1.4 ± 4.40 | 1.7 ± 2.54 | -1.2 ± 3.97 | -0.2 ± 3.13 | -0.4 ± 4.17 | -0.7 ± 6.18 |
| 30 | -1.4 ± 4.29 | 3.5 ± 6.52 | 0.3 ± 6.45 | 1.2 ± 3.27 | -2.1 ± 5.53 | 0.8 ± 5.24 |
| 60 | -1.3 ± 4.96 | 1.0 ± 5.79 | -0.8 ± 5.11 | 1.3 ± 4.13 | -0.6 ± 5.73 | 2.9 ± 5.80 |
| 120 | -1.8 ± 5.24 | 1.8 ± 4.43 | -4.1 ± 5.85 | -0.3 ± 2.32 | -2.4 ± 4.17 | -1.3 ± 4.63 |
| 240 | -2.7 ± 4.98 | -2.8 ± 6.80 | -3.0 ± 7.78 | -1.1 ± 5.53 | -2.1 ± 6.09 | 0.3 ± 3.85 |

**Figure C3: Mean Blood Pressure Change from Baseline ± SD, mmHg**

Judging from the small changes in mean BP, it appears that the changes in systolic and diastolic BP were directionally opposite but similar in magnitude, resulting in little change in mean BP. One possible explanation for this finding is that the pulse pressure was narrowed by the modest tachycardia that occurred early during and after the infusion. Figure C4 shows the chronology of change in the three blood pressure measurements and heart rate. The fluctuations in systolic and diastolic pressure correlate (on visual inspection) with the change in heart rate. A mixed-effects model with blood pressure change as the dependent variable, heart rate as the primary covariate, baseline heart rate as a fixed effect, and subject as a random effect showed a statistically significant correlation between change in heart rate and change in systolic BP (*P* = .0258), and a nearly significant relationship between change in heart rate and change in diastolic BP (*P* = .1066).

**Figure C4: Relationship between mean BP, systolic BP, diastolic BP, and heart rate (SD), 600 mg dose**

**Conclusions**

Though systolic blood pressure was modestly reduced at the end of the infusion of HBI-3000, this reduction was relatively small (-3.6 mmHg, when adjusted for placebo) and transient in nature, probably of little or no clinical relevance. While an effect of the drug on cardiac output, contractility, or vascular resistance cannot be excluded, the fact that mean blood pressure did not fall at the time of maximum exposure suggests that sulcardine had little or no hemodynamic effects at the tested dose levels.
